# Maternal lineage of Okinawa indigenous Agu pig inferred from mitochondrial DNA control region

**DOI:** 10.5713/ajas.18.0378

**Published:** 2018-10-26

**Authors:** Shihei Touma, Hirotoshi Shimabukuro, Aisaku Arakawa, Takuro Oikawa

**Affiliations:** 1 Okinawa Prefectural Livestock and Grassland Research Center, Nakijin, Okinawa 905-0426, Japan; 2 Okinawa Prefectural Livestock Breeding Center, Kunigami, Okinawa 905-1503, Japan; 3 Institute of Livestock and Grassland Science, National Agriculture and Food Research Organization (NARO), Tsukuba, Ibaraki 305-0901, Japan; 4 Faculty of Agriculture, University of the Ryukyus, Nishihara, Okinawa 903-0213, Japan

**Keywords:** Okinawa Indigenous Pig, Agu, Mitochondrial DNA, Maternal Lineage, Haplotype

## Abstract

**Objective:**

The Agu is the only native pig breed in Japan, which is reared in Okinawa prefecture, the southernmost region in Japan. Its origins are considered to be of Asian lineage; however, the genetic background of the Agu is still unclear. The objective of this study was to elucidate the maternal lineage of the Okinawa indigenous Agu pig with the use of the mitochondrial DNA (mtDNA) control region.

**Methods:**

The mtDNA control regions of Agu pigs were sequenced and the phylogenetic relationship among Agu, East Asian and European pigs was investigated with the use of 78 Agu individuals.

**Results:**

Twenty-seven polymorphic sites and five different haplotypes (type 1 to type 5) were identified within the Agu population. Phylogenetic analysis indicated that types 1 and 2 were included in East Asian lineages; however, the remaining types 3, 4, and 5 were of European lineages, which showed a gene flow from European pigs in the 20th century. Sixty-seven out of 78 Agu individuals (85.9%) possessed mtDNA haplotypes 1 and 2 of the East Asian lineage, which were identical to two haplotypes of ancient mtDNA (7,200 to 1,700 years before the present) excavated at archaeological sites in Okinawa.

**Conclusion:**

This study confirmed that the East Asian lineage is dominant in the maternal genetic background of the Agu population, supporting the hypothesis that the ancestors of the Agu pig were introduced from the Asian continent.

## INTRODUCTION

The Agu is the only native pig breed in Japan, which is reared in Okinawa prefecture, the southernmost region in Japan. Agu pigs are relatively small-bodied (around 100 kg at maturity) with coarse black hairs, semi-erect ears and a sagging abdomen. The reproductive performances of Agu sows are as follows: total number born, number born alive and number weaned was 4.8, 4.1, and 3.3, respectively which was smaller than half the number of European breeds [1]. Despite their low growth performance and lean meat productivity, Agu pigs have good meat qualities such as a lower cooking loss and higher intramuscular fat content than commercial pig breeds [2]. Meat of the Agu pig has been a source of food for the inhabitants of Okinawa islands [3]. The introduction of European breeds in the early 1900s for improving the performance of Agu pigs gradually reduced the number of pure Agu, which by 1980s stood at about 30 animals. Because of the continuous promotion of conservation and propagation by the local government, the population of Agu pigs has recovered and reached around 1,200 animals in 2016.

Although the details of the origins of the Agu are not yet understood, It is generally believed that the ancestors of the Agu pig were introduced from the Asian continent in the latter half of the 14th century when the ancient Kingdom of Okinawa began to trade with China [4,5]. Nonetheless, recent studies on ancient mitochondrial DNA (mtDNA) from bones excavated at archaeological sites have suggested that the introduction of domestic pigs to Okinawa from the Asian continent dates back to prehistoric times [6,7].

To date, the sequences of the mtDNA cytochrome b gene of Agu pigs have matched perfectly with those of Meishan and Jinhua pigs [8]. Phylogenetic analysis has disclosed that the only haplotype of Agu mtDNA discovered was clustered into an East Asian clade [9–11]; however, the investigations were conducted on a limited number of individuals. The genetic background of Agu pigs remains unclear; therefore, elucidating the genetic structure of the Agu is important for furthering its conservation and breeding. In this study, to clarify the maternal genetic background of Agu pigs, firstly, the mtDNA control regions of Agu pigs was sequenced and the phylogenetic relationship among Agu, East Asian and European pigs was investigated with the use of 78 Agu individuals as representative of the entire population. Secondly, the mtDNA control region of the currently modern Agu pigs was compared with that of ancient pigs excavated at archaeological sites in Okinawa.

## MATERIALS AND METHODS

### Animals

Seventy-eight Agu pigs were collected from 24 farms in Okinawa prefecture that maintain the Agu population. Since pedigree information on Agu pigs is incomplete at most farms, samples were collected from non sibling individuals so that the samples were as unrelated as possible. DNA was extracted from the auricle by the phenol-chloroform method. The sequences of the mtDNA control region from 30 pigs were obtained from the National Center for Biotechnology Information (NCBI) database (GenBank) (Table 1): 11 indigenous pigs from China, 4 pigs from Taiwan, 1 pig from Korea, 3 Japanese wild boars (*Sus scrofa leucomystax*), 5 Ryukyu wild boars (*Sus scrofa riukiuanus*) and 6 domestic pigs from Europe. Furthermore, the haplotypes of the Agu pig identified in this study were compared with five ancient mtDNA haplotypes unearthed at archaeological sites (7200–1700 years before the present; BP) on Okinawa Islands [6,7] (NCBI GenBank; Table 2). The five Ancient haplotypes have been classified into East Asian domestic pigs by phylogenetic analysis [6,7].

### Amplification and squencing of mtDNA control region

The mtDNA control region was amplified by polymerase chain reaction (PCR) using the following primers: mitM1 (5′-GG AGACTAACTCCGCCATCA-3′; nucleotide positions 15379–15398 of pig mtDNA sequence FJ237003) and mitM2 (5′-TT TTGGGGATGCTTAGACTCA-3′; positions 15995–16016). PCR was carried out on a volume of 50 μL, containing 40 ng DNA as a template, 10 mM Tris-HCl (pH 8.3), 1.5 mM MgCl_2_, 200 μM of each dNTP, 0.5 μM of each primer and 1 unit of Taq DNA polymerase. Amplification was carried out at 94°C for 9 min, for 10 cycles at 94°C for 30 s, 63°C for 30 s, and for 40 additional cycles at 94°C for 30 s, 60°C for 20 s and 72°C for 1 min. The amplified products were purified using ExoSAP-IT (Applied Biosystems, Tokyo, Japan) and sequenced with the BigDye Terminator v3.1 Cycle Sequencing Kit (Applied Biosystems, Japan) on a model 3130xl DNA sequencer (Applied Biosystems, Japan). The sequencing primers were mitM1 and mitM2, which were used for the PCR amplification. The full sequences of the mtDNA control region were assembled by overlapping forward and reverse sequencing products.

### Data analyses

The haplotypes of mtDNA control region of Agu pigs were aligned with the haplotypes of 30 pig breeds obtained from the NCBI database (Table 1). The common tandem repeat motif 5′-CGTGCGTACA-3′ and the specific repeat sequence motifs 5′-ACACAAACC-3′ and 5′-TAAAACACTTA-3′ in Lanyu pigs were excluded from the analysis [12]. Clustal W [13] was used to multiply-align these sequences. A phylogenetic tree was generated by the maximum-likelihood method based on the Hasegawa-Kishino-Yano model with invariable positions and Gamma distribution [14] using MEGA 7.0 with option ‘Find Best DNA Models’ [15]. The bootstrap method [16] from 1,000 bootstrap repetitions was used to assess the robustness of the phylogeny. A median-joining network was constructed using Agu haplotypes with NETWORK 5.0 [17].

## RESULTS

**Figure 1:**
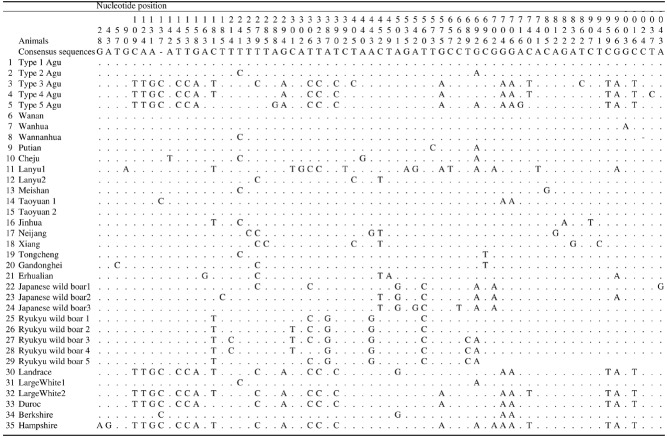
Nucleotide substitution sites of control region sequences of Agu pigs and 30 Asian and European pigs, and wild boars. Position 1 corresponds to the first base of the control region. The nucleotides identical to the mtDNA control region consensus sequences are denoted by dots (.). Dashes (-) indicate gaps.

Sixty-five polymorphic sites were observed within the sequences of the mtDNA control region, about 1,045 bp after exclusion of the repeat motif. Fifty-eight transitions, five transversions and two nucleotide gaps were obtained (Figure 1). Twenty-seven polymorphism sites were observed within the Agu population, demonstrating five different haplotypes (type 1-type 5). Haplotype 1 found in 23 individuals was completely identical to the sequence observed in Taoyuan 2 and Wanan pigs, the indigenous breeds of China and Taiwan, respectively. Haplotype 2, the major haplotype in the collected samples was found in forty-four Agu pigs. The remaining haplotypes 3, 4, and 5 were obtained from two, four and five Agu pigs, respectively.

**Figure 2:**
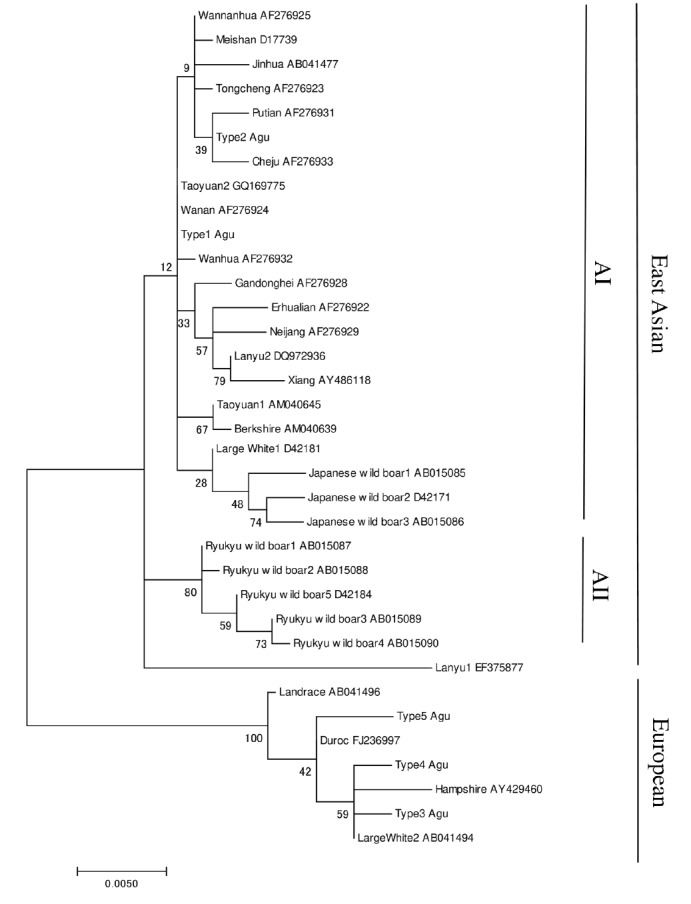
Maximum likelihood phylogenetic tree among the 35 pigs mtDNA control region haplotypes. The phylogenetic tree was inferred by the Hasegawa-Kishino-Yano model with invariable positions and Gamma distribution. Numbers on the branches show bootstrap values based on 1,000 bootstrap replicates.

**Figure 3:**
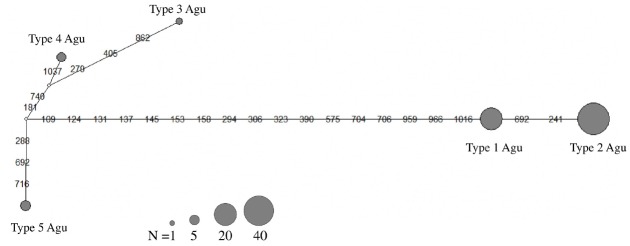
Median-Joining network of mtDNA control region haplotypes found in Agu pig population. The size of the gray circle is proportional to the frequency of the haplotype. White circles represent hypothetical haplotypes. Numbers on lines indicate the positions of substitutions.

The maximum likelihood phylogenetic tree was constructed based on the Hasegawa-Kishino-Yano model to infer the genetic relationship between Agu pigs, East Asian pigs, European pigs and wild boars (Figure 2). Thirty-five mtDNA haplotypes were divided into two major clades: East Asian and European (except for Berkshire and part of Large White breeds). The East Asian clade was divided into two subclades, AI and AII; the former included domestic pigs and Japanese wild boars, the latter comprised haplotypes of Ryukyu wild boars. Agu haplotypes 1 and 2 were clustered within the AI clade: Agu haplotype 1 was clustered within Taoyuan 2, Wanan and Wanhua pigs; haplotype 2 was clustered within the haplotype of Putian and Cheju pigs. The other haplotypes (3, 4, and 5) found in the Agu population were classified within the European type clade. Sixty-seven out of 78 Agu individuals (85.9%) possessed mtDNA haplotypes 1 and 2 of the East Asian lineage. A median-joining network of haplotypes was constructed to visualize the relationship among haplotypes within Agu population (Figure 3). Five haplotypes of the Agu were divided into two groups (type 1 and 2 vs type 3, 4, and 5), which is consistent with the maximum likelihood Phylogenetic tree (Figure 2).

**Figure 4:**
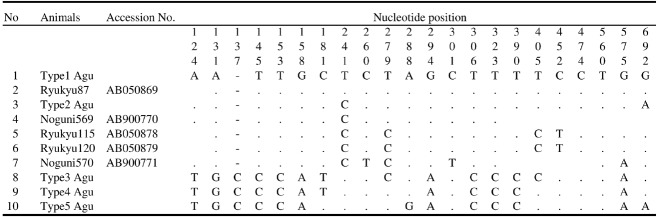
Nucleotide substitution sites of control region sequences in Agu pigs and Ancient pigs classified into East Asian domestic pigs by a phylogenetic analysis (Watanobe et al [6]; Takahashi et al [7]). The nucleotides identical to the sequences of type1 Agu are denoted by dots (.). Dashes (-) indicate gaps.

In comparing the haplotypes of Agu pigs with five haplo types of ancient pigs in Okinawa (Table 2), two haplotypes of ancient mtDNA were found identical to the Agu haplotypes in this study. One ancient mtDNA (Ryukyu 87 2000-1700 BP, 572 bp) was identical to Agu haplotype 1 and the other mtDNA (Noguni 569 7200-4400 BP, 215 bp) was identical to Agu haplotype 2 (Figure 4).

## DISCUSSION

The Agu is the only indigenous pig in Japan. We sequenced the complete mtDNA control regions of 78 Agu pigs from 24 farms as representative of the entire population. This is the first comprehensive survey of the mtDNA control region of the Agu pig population in which five haplotypes (type 1 to type 5) were detected. Haplotype 1 accorded with previous studies [9,11]. The other four haplotypes were newly found in the Agu pig population. These previous studies by Watanobe et al [9] and Takada et al [11] were conducted on a limited number of Agu pigs (one and six individuals from one farm). The present comprehensive survey with the use of 78 individuals from 24 farms provided four new haplotypes in the Agu pig population. In the phylogenetic analysis, 35 mtDNA haplotypes of Agu pigs, East Asian pigs, European pigs and wild boars were generally divided into two major clades: East Asian and European, as described in previous studies [10,18–20]. Agu mtDNA haplotypes 1 and 2 were clustered within the East Asian clade, whereas Agu haplotypes 3, 4, and 5 were clustered within the European clade (Figure 2). In addition, a median-joining network divided five haplotypes of the Agu into two groups (type 1 and 2 vs type 3, 4, and 5). These results indicated that the Agu pig population has two distinct maternal lineages: East Asian pigs and European pigs; Eastern Asian lineage is dominant in the Agu population. Based on historical background, the ancestors of the Agu are assumed to have been introduced from Asian continent during the 14th century [3,4]. In fact, their morphological features are similar to some of the Asian breeds. Furthermore, the sequence of Agu haplotype 1 was identical to Wanan and Taoyuan 2 pigs, the indigenous breeds of China and Taiwan, respectively (Figure 1). In 1904, Berkshire and Yorkshire breeds were introduced into Okinawa to improve the productivity of Agu pigs. In addition, after World War II many European breeds such as Hampshire, Chester White, Landrace, Large White and Duroc breeds were introduced into Okinawa [21,22]. In light of the history, Haplotypes 1 and 2 showed an East Asian lineage derived from the ancestors of the Agu pig. In contrast, the other three haplotypes (3, 4, and 5) showed European lineages were the result of a maternal gene flow from European breeds introduced into Okinawa after 1904.

The Ryukyu wild boar ( *Sus scrofa riukiuanus*) has inhabited the Okinawa islands since the Pleistocene [23]. In the present study, no haplotypes of the Ryukyu wild boar were found in Agu individuals. Moreover, phylogenetic analysis revealed that haplotypes of Agu pigs were distinct from those of Ryukyu wild boars (Figure 2). Our results indicated the Ryukyu wild boar is not the ancestor of Agu pigs, and suggested that no maternal gene flow took place from the Ryukyu wild boar to the Agu pig population.

There are no historical records describing when the first domestic pigs were introduced into Okinawa. The earliest historical record about domestic pigs in Okinawa is found in the records of the Chosun Dynasty of Korea. The Annals of the Chosun Dynasty showed that Korean castaways who stayed in Okinawa for several months at the end of the 15th century saw domestic pigs. Although the exact time period is unclear, it is believed that the first introduction of domestic pigs in Okinawa was in the 14th century when the ancient Kingdom of Okinawa began to trade with China [4,5]. Nonetheless, ancient mtDNA haplotypes of the East Asian domestic pig lineage excavated at archaeological sites (2000-1700 and 7200-4400 BP, respectively) have been found in Okinawa [6,7], suggesting that domestic pigs were introduced into Okinawa from the Asian continent long before the commonly held view. In this study, two haplotypes (1 and 2) of the East Asian lineage were found in Agu pigs. Agu haplotype 1 has been reported as identical to an ancient mtDNA haplotype of Ryukyu 87 (AB050869) excavated at an archaeological site (2000-1700 BP) [6]. Our study, on the other hand, demonstrated that the new Agu haplotype 2 was identical to Noguni 569 (AB900770) found at an archaeological site (7200-4400 BP) by Takahashi et al [7] (Figure 4). These results revealed the possibility that the mtDNA sequences of ancient pigs have been inherited by the modern Agu pig population. We are not able to come to that conclusion because of the short sequences of ancient mtDNA. In order to understand more clearly the relation between modern Agu pigs and ancient pigs in Okinawa, further analyses are needed, especially with the use of longer sequences of ancient mtDNA.

In conclusion, our study demonstrated that the Agu pig population has East Asian and European maternal lineages, that most Agu individuals possess East Asian haplotypes, and that East Asian lineage haplotypes of Agu pigs was derived from ancestral pigs introduced during the 14th century, or earlier, whereas European haplotypes are the result of maternal gene flow from European breeds introduced into Okinawa after 1904. Our results supported the hypothesis that the ancestors of the Agu pig were introduced from the Asian continent. The present study also provided useful information for further conservation of the Agu pig as the only native pig in Japan. However, mtDNA doesn’t reflect the characteristics of the nuclear genome, as it gives only maternal genetic information. Therefore, further molecular studies using nuclear genome markers are required for future management of this native pig.

## Figures and Tables

**Table 1:** Haplotypes of mtDNA control region from other pig breeds

No.	Animals	Country	Accession no.
1	Wanan	China	AF276924
2	Wanhua	China	AF276932
3	Wannanhua	China	AF276925
4	Putian	China	AF276931
5	Meishan	China	D17739
6	Jinhua	China	AB041477
7	Neijang	China	AF276929
8	Xiang	China	AY486118
9	Tongcheng	China	AF276923
10	Gandonghei	China	AF276928
11	Erhualian	China	AF276922
12	Cheju	Korea	AF276933
13	Lanyu 1	Taiwan	EF375877
14	Lanyu 2	Taiwan	DQ972936
15	Taoyuan 1	Taiwan	AM040645
16	Taoyuan 2	Taiwan	CQ169775
17	Japanese wild boar1	Japan	AB015085
18	Japanese wild boar2	Japan	D42171
19	Japanese wild boar3	Japan	AB015086
20	Ryukyu wild boar 1	Japan	AB015787
21	Ryukyu wild boar 2	Japan	AB015788
22	Ryukyu wild boar 3	Japan	AB015789
23	Ryukyu wild boar 4	Japan	AB015790
24	Ryukyu wild boar 5	Japan	D42184
25	Landrace		AB041496
26	Large White1		AB041499
27	Large White2		AB041494
28	Duroc		FJ236997
29	Berkshire		AM040639
30	Hampshire		AY429460

**Table 2:** Haplotypes of mDNA control region of ancient pigs excavated from archaeological sites on Okinawa islands

No.	Haplotype	Date	Accession no.	Nucleotide positions (bp)	Reference
1	Ryukyu87	2000-1700 BP	AB050869	131-704	Watanobe et al [6]
2	Ryukyu115	2000-1700 BP	AB050878	131-704	Watanobe et al [6]
3	Ryukyu120	2000-1700 BP	AB050879	131-704	Watanobe et al [6]
4	Noguni569	7200-4400 BP	AB900770	111-326	Takahashi et al [7]
5	Noguni570	7200-4400 BP	AB900771	111-326, 540-704	Takahashi et al [7]
